# National trends in age-standardized mortality attributable to hypertension in Peru

**DOI:** 10.1590/2175-8239-JBN-2020-0009

**Published:** 2021-01-11

**Authors:** Percy Herrera-Añazco, Noé Atamari-Anahui, Maycol Suker Ccorahua-Rios, Elard Amaya

**Affiliations:** 1 Universidad Señor de Sipán, Chiclayo, Peru.; 2 Universidad San Ignacio de Loyola, Vicerrectorado de Investigación, Unidad de Investigación para la Generación y Síntesis de Evidencias en Salud, Lima, Perú.; 3 Instituto Nacional de Salud del Niño-Breña, Asociación de Médicos Residentes del Instituto Nacional de Salud del Niño (AMERINSN), Lima, Perú.; 4 Universidad Nacional de San Antonio Abad del Cusco, Escuela Profesional de Medicina Humana, ASOCIEMH CUSCO, Cusco, Perú.; 5 Universidad San Ignacio de Loyola, Vicerrectorado de Investigación, Centro de Excelencia en Investigaciones Económicas y Sociales en Salud, Lima, Perú.; 6 Hospital Nacional 2 de mayo, Lima, Peru.

**Keywords:** Mortality, Hypertension, Public Health, Universal Health Insurance, Peru., Mortalidade, Hipertensão, Saúde Pública, Cobertura Universal do Seguro de Saúde, Peru.

## Abstract

**Introduction:**

Hypertension (HTN) is a public health problem. The prevalence and mortality rates are significantly higher in middle and low-income countries, such as Peru. This study aimed to determine the trend of mortality attributable to HTN for the 2005-2016 period in Peru.

**Methods:**

We conducted a secondary analysis based on death certificates provided by the Ministry of Health. We applied linear regression models to test the HTN mortality rate trend.

**Results:**

The age-standardized HTN mortality per 100,000 inhabitants decreased from 14.43 for the 2005 to 2010 period to 11.12 for the 2011 to 2016 period. The coast was the natural region with the highest decrease in mortality rate. Moreover, Tumbes, Callao, and Lambayeque were regions with the highest decline in mortality rate.

**Conclusion:**

The age-standardized mortality attributable to HTN decreased in Peru, with variations in both natural and political regions of the country.

## Resumo

**Introdução:**

A hipertensão arterial (HA) é um problema de saúde pública. As taxas de prevalência e mortalidade são significativamente mais altas em países de média e baixa renda, como o Peru. O objetivo do estudo foi determinar a tendência de mortalidade atribuível à HA para o período de 2005-2016 no Peru.

**Métodos:**

Realizamos uma análise secundária com base em atestados de óbito fornecidos pelo Ministério da Saúde. Aplicamos modelos de regressão linear para testar a tendência da taxa de mortalidade por HA.

**Resultados:**

A mortalidade por HA padronizada para idade por 100.000 habitantes diminuiu de 14,43 no período de 2005 a 2010 para 11,12 no período de 2011 a 2016. O litoral foi a região natural com maior queda na taxa de mortalidade. Além disso, Tumbes, Callao e Lambayeque foram as regiões com maior declínio na taxa de mortalidade.

**Conclusão:**

A mortalidade padronizada para idade atribuível à HA diminuiu no Peru, com variações tanto em regiões naturais como políticas do país.

## Introduction

Hypertension (HTN) is a public health problem.^1^^,^ 2 The number of hypertensive patients will increase by 60%, with an estimated 1.65 trillion cases worldwide in the next 25 years.^1^ In middle and low-income countries, the prevalence of HTN is greater than in high-income countries. Likewise, the healthcare cost is higher in this countries and occur approximately 80% of the cardiovascular events associated with HTN in the world.^2^^,^ 3 ^,^ 4

According to the Global Burden Disease (GBD), mortality associated with systolic blood pressure higher than 140 mmHg increased from 97.9 to 106.3 deaths per 100,000 population globally. In the Andean region-which includes developing countries such as Peru, Ecuador, and Bolivia-mortality associated with HTN increased from 10.5 to 28.5 deaths per 100,000 population between 1990 and 2015.^2^ However, this trend may not be the same in each of these countries, given the substantial differences in their health systems.^5^

In Peru, although there are studies that have assessed the prevalence and other aspects related to HTN,^6^^,^ 7 mortality has been less studied. Therefore, the objective of this study was to determine the mortality trend attributable to HTN in patients of the Ministry of Health (MINSA, by its Spanish acronym) for the period 2005-2016 at the national and regional levels.

## Methods

### Study design and information source

A descriptive study with trend analysis was conducted. The units of analysis were the 25 political regions of Peru: 24 regions and a constitutional province, as well as its three natural regions: Coast, Highlands, and Jungle. The source of information was the annual death records, based on death certificates for the 2005-2016 period, provided by the MINSA. This database contains all deaths registered in death certificates of the country. Patients who did not belong to the MINSA were excluded.

### Procedures

The database with information on death cases from a basic cause was requested through the MINSA Public Information Access Platform (http://www.minsa.gob.pe/portada/transparencia/solicitud/frmFormulario.asp). In Peru, the death certificate registers three types of causes of death: direct, intermediate, and basic. For this study, we consider the basic cause of death. This is defined as the disease that initiates the chain of pathological events that led directly to death.^8^

### Variables

The outcome variable was the mortality attributable to hypertension, which was calculated as a ratio between the annual number of deaths registered (2005-2016 period) and the population of each region, expressed per 100,000 inhabitants. These variables were obtained from the MINSA database ICD 10: I10 coding and were evaluated by year, sex, age groups, natural regions, and political regions. The population for each region-year of the study was obtained from the website of the National Institute of Statistics of Peru (https://www.inei.gob.pe/estadisticas/indicetematico/population-estimates-and-projections/). In addition, standardized mortality by age was obtained through the direct method, using the population of the World Health Organization 2000-2025 as a reference.^9^

### Analysis of data

The analysis was performed using the statistical package STATA^(r)^ 15.0 (StataCorp, College Station, TX, USA). We used mean and standard deviation to describe numeric variables and absolute and relative frequencies for categorical variables. To determine the mortality trend attributed to HTN we calculated the percentage change in mortality from previous studies.^10^^,^ 11 ^,^ 12 First, we averaged mortality rates of the first six and last six years evaluated, to reduce the measurement error bias associated with taking a single year as a reference. Then, we calculate the difference in the mortality rate for the 2011-2016 period (t2) and 2005-2010 (t1) period, and then the percentage of change was calculated as: ((t2-t1) / t1) x 100.

Likewise, linear regression models were applied, in which the outcome variable was the age-standardized mortality attributed to HTN and the explanatory variable was time, which sought to assess the trend of mortality attributable to HTN for the period of analysis. In these models, 95% confidence intervals (95% CI) with errors corrected for robust variance were calculated and statistically significant coefficients were considered with a *p* < 0.05 value and marginally significant, with a *p* < 0.1 value.

### Ethical aspects

This study performed an analysis of secondary data that was obtained through a request to a public access website and published reports.

## Results

During the 2005-2016 period, the MINSA database recorded 33,405 deaths attributed to HTN, of which 16,871 cases (50.5%) were female. Moreover, 29,229 cases (87.5%) corresponded to people over 60 years old, 3,893 (11.7%), to the 30-59 age group, and 283 cases (0.8%), to people under 30 years old.

**Figure 1:**
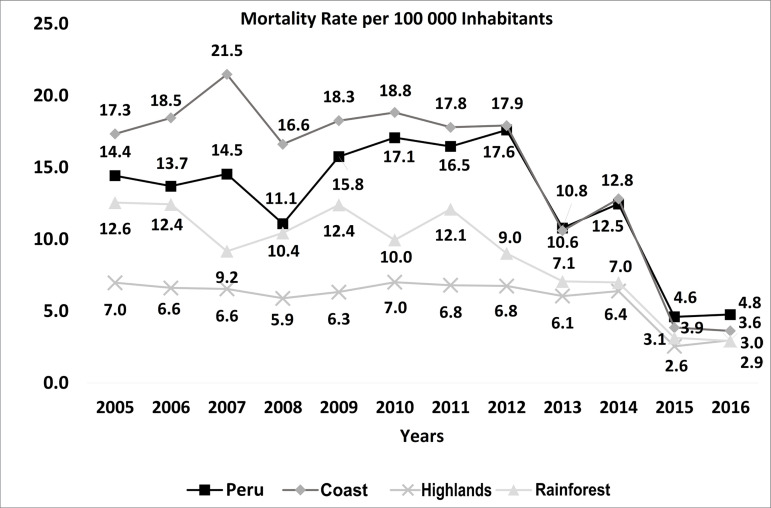
Age-standardized mortality rates attributable to hypertension registered in the Ministry of Health of Peru in the 2005-2016 period.

The age-standardized mortality rate attributed to HTN per 100,000 inhabitants decreased from 14.43 for the 2005 to 2010 period, to 11.12 for the 2011 to 2016 period (Figure 1). Natural regions with the greatest decrease in mortality were coast (% change = -39.97), followed by rainforest (% change = -38.38). Political regions with higher decrease in mortality attributed to HTN were Tumbes (% change = -77.35), Loreto (% change = -70.96), and Ucayali (% change = -68.88), however, some regions presented significant increase such as Ica (% change = 184.08) and Madre de Dios (% change = 136.53) (Table 1).

**Table 1:** Age-standardized mortality rates attributable to hypertension in the Ministry of Health of Peru by regions

Regions	Standardized mortality x 100 000 inhabitants			Coef B (95%CI)	p value
	2005-2010 (t1)	2011-2016 (t2)	% change ((t2-t1)/t1)*100		
**Peru (Country)**	14.43	11.12	-22.98	-0.70 (-1.36 to -0.03)	0.043
**Coast**	18.50	11.10	-39.97	-1.31 (-2.07 to -0.55)	0.003
**Andean**	6.57	5.26	-19.91	-0.27 (-0.51 to -0.03)	0.028
**Jungle**	11.16	6.88	-38.38	-0.81 (-1.11 to .0.49)	< 0.001
Amazonas	7.56	6.67	-11.80	-0.40 (-0.92 to 0.11)	0.109
Ancash	8.96	6.09	-32.04	-0.49(-1.22 to 0.24)	0.165
Apurímac	7.20	6.14	-14.77	-0.28 (-0.92 to 0.35)	0.345
Arequipa	2.42	2.55	5.66	0.01 (-0.12 to 0.14)	0.001
Ayacucho	2.93	2.20	-24.77	-0.08 (-0.15 to -0.11)	0.027
Cajamarca	11.80	12.32	4.41	0.03 (-0.32 to 0.39)	0.853
Callao	32.98	16.11	-51.17	-2.69 (-4.30 to -1.07)	0.004
Cusco	2.87	2.05	-28.55	-0.15 (-0.37 to 0.05)	0.128
Huancavelica	7.48	7.51	0.40	-0.43 (-1.21 to 0.35)	0.250
Huánuco	7.35	4.21	-42.71	-0.51 (-0.97 to -0.04)	0.036
Ica	3.62	10.29	184.08	0.69 (-0.31 to 1.69)	0.156
Junín	6.89	3.79	-45.01	-0.35 (-0.94 to 0.24)	0.219
La Libertad	14.21	13.18	-7.22	-0.62 (-1.61 to 0.37)	0.196
Lambayeque	26.16	14.28	-45.40	-2.31 (-3.43 to -1.20)	0.001
Lima	10.21	6.66	-34.81	-0.62 (-1.09 to -0.13)	0.018
Loreto	8.86	2.57	-70.96	-1.05 (-1.96 to -0.14)	0.029
Madre de Dios	4.32	10.21	136.53	0.43 (-0.12 to 0.98)	0.109
Moquegua	11.42	8.65	-24.25	-0.65 (-1.29 to -0.01)	0.048
Pasco	9.61	6.23	-35.17	-0.73 (-1.19 to -0.27)	0.006
Piura	21.35	16.37	-23.34	-0.74 (-2.27 to 0.79)	0.310
Puno	4.74	4.77	0.56	-0.08 (-0.39 to 0.23)	0.569
San Martín	17.91	9.59	-46.43	-1.37 (-2.04 to -0.71)	0.001
Tacna	6.44	5.32	-17.46	-0.28 (-0.88 to 0.32)	0.327
Tumbes	40.08	9.08	-77.35	-4.60 (-5.95 to -3.25)	˂ 0.001
Ucayali	17.16	5.34	-68.88	-1.62 (-2.31 to -0.93)	˂ 0.001

The linear regression analysis showed a decrease in the national trend (β = -0.70; *p* = 0.043), and in its three natural regions. At regional level, the trend of greatest decrease was Tumbes (β = -4.60; *p* = ˂ 0.001), Callao (β = - 2.69; *p* = 0.004), and Lambayeque (β = -2.31; *p* = 0.001) (Table 1).

## Discussion

Our main results show a decrease in HTN mortality in the 2005-2016 period, with the coastal region having a greater decrease. Similarly, the region of Tumbes, Callao, and Lambayeque showed decreasing trends.

The decrease in the mortality trend attributable to HTN is inverse to that reported both worldwide and in the Andean region.^2^ However, unlike deaths reported in the GBD, our mortality attributable to HTN is determined by death certificates records and not by a systolic pressure higher than 140 mmHg. Therefore, it is possible that an under-registration of HTN be one of the explanations for this apparent decrease in mortality. On the other hand, it can also be a consequence of an improvement in health insurance coverage.^13^ In fact, health coverage increased from 34% to 47% in the 2009-2017 period in MINSA establishments,^13^ which is relevant since health systems coverage influences HTN results.^14^ Although there are no specific studies of this association, the increase in health insurance coverage could partially explain the improvement in the self-knowledge and control of HTN in Peru,^9^ which could have influenced the decrease in mortality attributable to HTN.

At the regional level, Tumbes, Loreto, Callao, and Lambayeque are regions where health insurance coverage is higher than the national average coverage rate (86.9%, 86.3%, 78.6%, and 78.5%, respectively), and Madre de Dios and Ica are regions with lower coverage rate (67.1% and 66.4%, respectively).^15^ This could explain the variations in mortality attributable to HTN found in these regions. Likewise, being the regions of the coast could explain the improvement.

The improvement of health insurance coverage increased access to antihypertensive drugs. The population that accessed this treatment at a national level increased from 60.3% to 63.9% in the years 2014-2017.^16^^,^ 17 Similarly, increased access to antihypertensive drugs could explain the surprising decrease in mortality from 2014. Likewise, improvement in the quality of records and information systems in Peru in recent years may mean that this information in previous years was possibly overestimated.^18^

The improvement in access to hypertensive treatment was highest in the coast and jungle regions, which could also explain the decrease in mortality rates in these natural regions.^16^^,^ 17 Although there was a decrease in access to antihypertensive treatment between 2016 and 2017 among the population of the coast, the access continued to improve in the jungle and andean regions.^16^ These changes must be analyzed by health policymakers.

Our study had some limitations. First, since it is a study that used a secondary data source, there could be errors or incomplete filling of death certificates, despite being filled by health staff. Second, it is possible that the number of deaths attributed to HTN are underestimated by collecting only the patients who went to MINSA establishments. Third, this study sought to describe the mortality attributed to HTN, although it did not describe the exact cause of death in patients whose underlying pathology was HTN. Finally, these results cannot be extrapolated to patients belonging to other health systems.

In conclusion, the mortality attributable to HTN has decreased among MINSA patients. This trend was highest for the coastal region, which include Tumbes, Callao, and Lambayeque.
